# Structure and biodiversity of coralligenous assemblages dominated by the precious red coral *Corallium rubrum* over broad spatial scales

**DOI:** 10.1038/srep36535

**Published:** 2016-11-18

**Authors:** Edgar Casas-Güell, Emma Cebrian, Joaquim Garrabou, Jean-Baptiste Ledoux, Cristina Linares, Núria Teixidó

**Affiliations:** 1 Institut de Ciències del Mar (ICM-CSIC), Passeig Marítim de la Barceloneta 37-49, 08003 Barcelona, Spain; 2 Centre d’Estudis Avançats de Blanes (CEAB-CSIC), Accés Cala Sant Francesc 14, 17300 Blanes, Girona, Spain; 3 Departament de Ciències Ambientals, Facultat de Ciències, Universitat de Girona, Girona, Spain; 4 UM110, CNRS/INSU, IRD, Aix-Marseille Université, Université du Sud Toulon Var, Mediterranean Institute of Oceanography (MIO), Marseille, France; 5 CIMAR/CIIMAR, Centro Interdisciplinar de Investigaçao Marinha e Ambiental, Universidade do Porto, Rua dos Bragas 177, 4050-123 Porto, Portugal; 6 Departament de Biologia Evolutiva, Ecologia i Ciències Ambientals, Facultat de Biologia, Universitat de Barcelona, Avinguda Diagonal 643, 08028, Barcelona, Spain; 7 Stazione Zoologica Anton Dohrn, Villa Dohrn-Benthic Ecology Center, Punta San Pietro, Ischia, Naples 80077, Italy

## Abstract

Data on species diversity and structure in coralligenous outcrops dominated by *Corallium rubrum* are lacking. A hierarchical sampling including 3 localities and 9 sites covering more than 400 km of rocky coasts in NW Mediterranean, was designed to characterize the spatial variability of structure, composition and diversity of perennial species inhabiting coralligenous outcrops. We estimated species/taxa composition and abundance. Eight morpho-functional groups were defined according to their life span and growth to characterize the structural complexity of the outcrops. The species composition and structural complexity differed consistently across all spatial scales considered. The lowest and the highest variability were found among localities (separated by >200 km) and within sites (separated by 1–5 km), respectively supporting differences in diversity indices. The morpho-functional groups displayed a consistent spatial arrangement in terms of the number, size and shape of patches across study sites. These results contribute to filling the gap on the understanding of assemblage composition and structure and to build baselines to assess the response of this of this highly threatened habitat to anthropogenic disturbances.

Fine-scale and high-resolution knowledge on the variability of the structure and functioning of key habitats over a wide range of spatial scales is important for effective management and conservation of coastal marine habitats^1^,^2^,^3^,^4^. Coralligenous outcrops foster some of the richest assemblages found in Mediterranean, harboring approximately 10% of marine Mediterranean species^1^,^5^,^6^,^7^. Most of the species characterizing these assemblages are long-lived algae and sessile invertebrates, which exhibit low dynamics and belong to various taxonomic groups such as sponges, corals, bryozoans and tunicates^1^,^8^,^9^. Coralligenous outcrops are hard bottoms of biogenic origin that are mainly produced by the accumulation of calcareous encrusting algae growing at low irradiance levels^1^. Nevertheless, rather than a single and uniform habitat, coralligenous biogenic formations comprise of a complex of different habitats whose occurrence is mainly determined by light exposure. As a result, coralligenous habitats can be dominated by calcareous algae to others completely dominated by macroinvertebrates with almost no algal growth. This mosaic of different habitats makes coralligenous outcrops highly diverse exhibiting great structural complexity^1^,^8^,^10^. Coralligenous outcrops are affected by several consequences of global change such as nutrient enrichment, invasive species, increase of sedimentation, mechanical impacts, mainly from fishing activities, as well as climate change^10^,^11^,^12^,^13^,^14^.

The precious Mediterranean red coral, *Corallium rubrum* (L. 1758), is one of the habitat-forming species structuring coralligenous outcrops^15^,^16^. This species is typically associated with animal dominated communities growing in dim light habitats, such as caves, vertical cliffs and overhangs, between 10 and 200 m in depth. The main threat to the red coral is intensive historical harvesting, which causes an overall shift in the population structure, resulting in a decrease in both biomass and colony size^17^,^18^,^19^. Climate warming and the potential effects of ocean acidification are also major threats affecting populations^12^,^20^,^21^,^22^. It has been demonstrated that a decrease in the abundance of habitat-forming species leads to a rapid fragmentation in community structure and a loss of species benefiting from the structural complexity these species provide^23^,^24^,^25^,^26^. *C. rubrum* is a slow-growing, long-lived species, and plays a key role as a habitat forming species and in the functioning of coralligenous habitats mainly due to its trophic activity, biomass and perennial biogenic structure as other Mediterranean gorgonian species^10^,^24^,^27^. Despite this essential role, few studies deal with the characterization and variability, at high resolution of the whole assemblage and over relevant temporal and regional scales (but see refs 28 and 29).

To our knowledge, few studies addressing coralligenous assemblages have extended their scope to larger spatial scales of up to >400 km of coastline^30^, and the majority focuses on the phytobenthic component^31^. Due to this lack of baseline data, the structure of coralligenous outcrops is poorly understood, preventing a proper assessment of its current state of biodiversity and the potential impacts of harvesting, and other disturbances related to global change, on red coral assemblages (but see ref. 4). Structural complexity of landscapes is commonly used to track changes in structure and dynamics in terrestrial ecosystems^32^. In addition, a landscape ecology approach has been successfully applied to marine benthic communities, providing new insights into the structural and ecological processes^33^,^34^,^35^. Here, structural complexity was assessed by applying landscape pattern indices based on the characteristics of patch mosaics (e.g. number, size and complexity of patches). This analysis considers benthic communities as patch mosaics corresponding to different categories (e.g. species).

Accurate, high resolution and large-scale biodiversity datasets are a basic resource that furnishes the essential information needed to promote sound conservation actions^36^. Focusing on a regional scale, the present study provides fine-scale, high-resolution quantification of the different components of biodiversity of coralligenous assemblages dominated by *C. rubrum*. These components are intended to give future assessments on the conservation status, as well as guide the development of a monitoring scheme for the rich coralligenous assemblages.

## Results

### Species composition

A total of 112 macrobenthic taxa were identified across the region studied: 20 macroalgae, 1 protozoan, 41 sponges, 6 hydrozoans, 11 anthozoans, 1 mollusk, 3 polychaetes, 21 bryozoans and 8 tunicates (see Supplementary Table S2) for the species list and assignment to the various morpho-functional groups). Of these taxa, 81 were perennial and 31 seasonal. Perennial taxa represented between 30 and 55% cover, whereas seasonal species barely reached 10% cover in all sites (Supplementary Figure S2). As expected, the red coral was one of the most abundant species. Colonies densities (number of colonies/0.32 m^2^) ranged from 13 ± 3.23 in Palazzinu (CorPlu) to 177 ± 4.48 in Plane Grotte Pérès (ProPer) (see Supplementary Table S9).

### Structural complexity

#### Morpho-functional groups. Number of species and % cover

**Figure 1:**
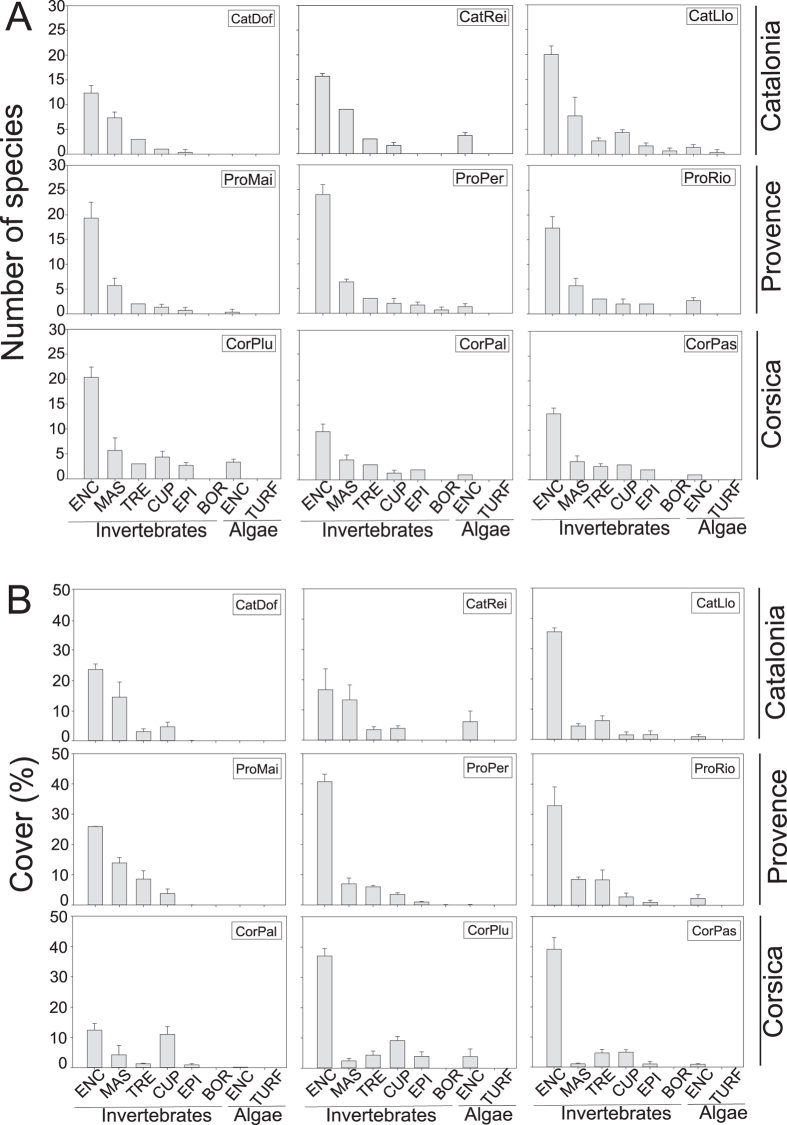
Diversity and abundance. Number of perennial species ± SD (**A**) and % of Cover ± SD (**B**) of each morpho-functional group and study site. ENC: encrusting; MAS: massive; TRE: tree; CUP: cup; BOR: boring; TURF: turf.

The number of species and % cover for each morpho-functional group (hereafter MFG) was similar (F_2,26_ = 1.63, p > 0.05 and F_2,26_ = 1.48, p > 0.05) among localities but showed significant differences (F_6,26_ = 8.95, p < 0.05 and F_6,26_ = 12.54, p < 0.05) among sites (Fig. 1a,b; Supplementary Table S4). The main group in terms of number of species and % cover was Animal encrusting, with values (hereafter, mean ± SD) ranging from 10 ± 2 to 24 ± 2 species in Palazzu (CorPal) and Plane Grotte Pérès (ProPer), respectively and % cover ranging from 10.91% ± 1.81 to 39.81% ± 3.26 in Palazzu (CorPal) and Passe Palazzu (CorPas), respectively. The second MFG characterizing the assemblage in Catalonia and Provence, in terms of number of species and % cover, was Animal massive with % cover ranging from 3.96% ± 0.47 to 14.76% ± 4.21 in Passe Palazzu (CorPas) and Cova Dofí (CatDof), respectively. In Corsica, Animal massive was the second group in terms of number of species, but the second most abundant group in terms of % cover was Animal cup. Animal tree was, in general, the less abundant MFG in terms of species number (ranging from 1 ± 0 to 4 ± 1 species) with low to moderate % cover since we only took into account the basal parts of colonies (ranging from 1.58% ± 0.48 to 8.68% ± 2.21 in Palazzu (CorPal) and Maïre Grotte (ProMai). The remaining categories Algal encrusting and turf and Animal epibiont and Boring were represented by very few species and showed the lowest % cover values (Fig. 1a).

### Species composition

**Figure 2:**
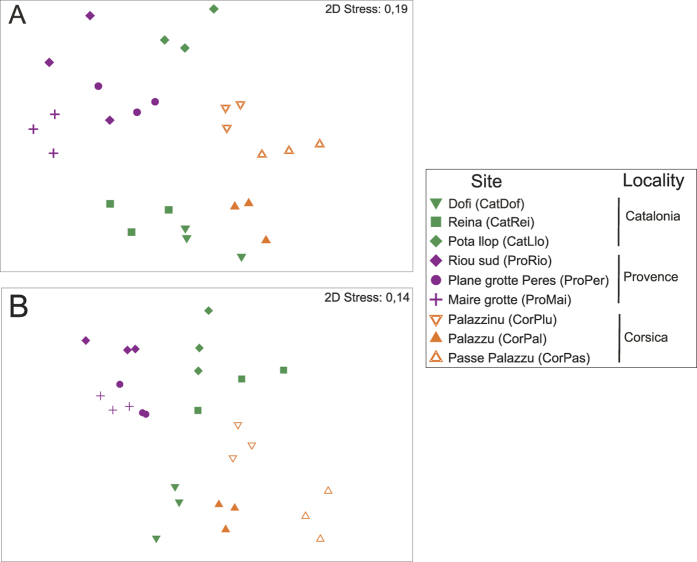
Non-metric multidimensional scaling (nMDS) ordination plot of perennial macrobenthic species in the three localities of the NW Mediterranean Sea. Analysis performed on Bray-Curtis dissimilarities for (**A**) presence-absence data and (**B**) % of cover. For each locality (orange = Corsica; purple = Provence; green = Catalonia), the three sites are shown by different shapes.

The structure and composition of perennial species assemblages differed significantly among sites (F_6,26_ = 7.02, p < 0.05 and F_6,26_ = 7.79, p < 0.05) as well as among localities (F_2,26_ = 2.45, p < 0.05 and F_2,26_ = 7.02, p < 0.05) regardless of the parameter analyzed (presence/absence and % cover, Fig. 2; Supplementary Table S5). Accordingly, the variability for each of the spatial factors (*Site* and *Locality*) showed a consistent pattern: the highest percentage of variation was found at *Site* spatial level (values ranging from 21.14% to 35.07%), followed by *Locality* (values from 15.85% to 27.43%) and finally the residuals, at the sampling unit level (MSA), which showed the lowest source of variability (values from 14.92% to 23.32%). The estimates of variance components presented higher values for % cover than for presence/absence (Supplementary Table S5).

The SIMPER analysis (Tables 1 and 2 shows the number of species contributing more than 50% of the dissimilarity and similarity) showed an overall average dissimilarity (up to 75%) in % cover across localities, with values of 76.78% between Provence and Corsica, 68.38% between Corsica and Catalonia and 66.01% between Provence and Catalonia. Focusing on the Animal encrusting differences, the relative abundance of the sponges *Crella (Grayella*) *pulvinar*, *Pleraplyssilla spinifera*, *Dendroxea lenis* and the bryozoan *Gregarinidra gregaria* mainly explained the dissimilarities among localities. Regarding *Animal massive*, dissimilarities among localities were mainly explained by differences in the relative abundance of *Oscarella sp*. and *Petrosia ficiformis*.

### Diversity indices

**Figure 3:**
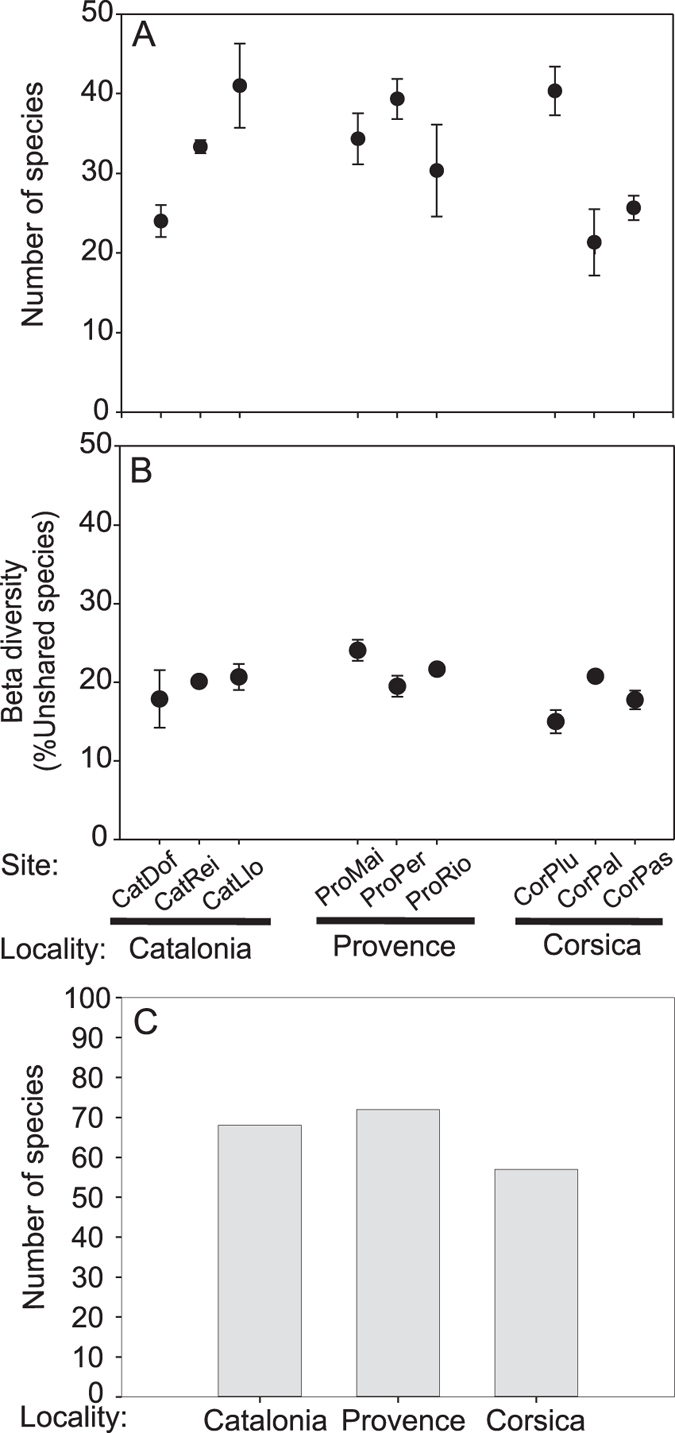
Diversity indices for all study sites and localities. (**A**) Number of species (alpha diversity), (**B**) % for unshared species (β-diversity) and (**C**) local number of species (gamma diversity).

The mean ± SD values of alpha diversity remained similar (F_2,26_ = 0.40, p > 0.05) for all localities but showed significant differences (F_6,26_ = 18.63, p < 0.05) among sites (Fig. 3a; Supplementary Table S3). Alpha diversity showed the highest variability in Catalonia and Corsica (Fig. 3a) with mean values ranging from 24 ± 2 to 41 ± 5.3 and 21 ± 4.2 to 40 ± 3.1, respectively. The variability in Corsica was due to Palazzinu (CorPlu), which showed the highest values for alpha compared with Palazzu (CorPal) and Passe Palazzu (CorPas). Provence presented consistent mean alpha diversity values at all sites ranging from 30 ± 5.8 to 39 ± 2.5 (Fig. 3a). The percentage of unshared species (beta diversity) was similar at both spatial levels (Fig. 3b; Supplementary Table S3). A similar pattern of beta diversity was found at all study sites with average percentage of unshared species ranging from 14.93% ± 1.5 to 24% ± 0.38 (Fig. 3b). Gamma diversity showed similar values among the three localities studied with 57 species in Corsica, 68 in Catalonia and 72 species in Provence (Fig. 3c). Values of all diversity indices, for all localities and sites, can be found in Supplementary Table S3.

### Landscape pattern indices

Overall, the mean perennial species cover was quite different between sites and across localities (F_2,26_ = 1.63, p > 0.05 and F_2,26_ = 1.48, p > 0.05), except for the site Palazzu (CorPal), which had the lowest value of 30% ± 1.82. Overall, values ranged from 60% ± 2.65 in Palazzinu (CorPlu) to 30% ± 1.82 in Palazzu (CatPal) (see Supplementary Figure S2).

For perennial species as a whole, number of patches (NP), mean patch size (MPS) and mean shape index (MSI) were similar among localities (F_2,107_ = 1.79, p > 0.05; F_2,107_ = 1.19, p > 0.05 and F_2,107_ = 0.56, p > 0.05) and sites (F_7,107_ = 0.64, p > 0.05; F_7,107_ = 0.28, p > 0.05 and F_7,107_ = 0.84, p > 0.05) (Supplementary Figure S3; Supplementary Table S7). The NP range from 802 to 1491 in Catalonia, from 826 to 1876 in Provence and from 914 to 1695 in Corsica. The MPS showed relatively small values ranging from 83 to 172 mm^2^ in Catalonia, from 87 to 196 mm^2^ in Provence and from 80 to 165 mm^2^ in Corsica. The MSI showed a narrow range over all the localities, with values from 0.37 to 0.57.

**Figure 4:**
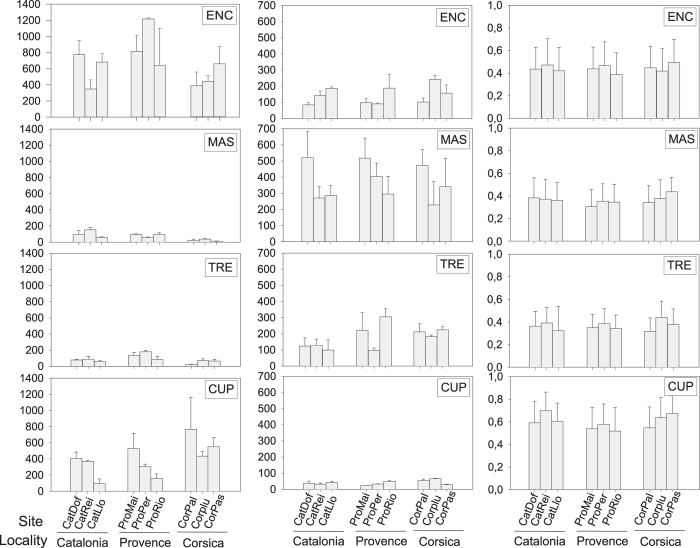
Landscape pattern indices. Number of patches (first column), Mean patch size (second column) and Mean shape index (third column) of each invertebrate morphofunctional group at each site studied. ENC: encrusting, MAS: massive, TRE: tree, CUP: cup.

Within perennial species, a high NP for Animal encrusting characterized benthic seascape (values ranging from 351 ± 114 to 1221 ± 14). This group was also the most important in terms of % cover, with values ranging from 353.30 mm^2^ ± 92.90 to 1240.33 mm^2^ ± 10.65 while displaying relatively small MPS (values ranging from 84.60 mm^2^ ± 13.83 to 240.61 mm^2^ ± 25.08) at all localities and sites (Fig. 4; Supplementary Table S8). Animal encrusting patches were quite irregular with MSI values close to 0.4 at all localities and sites (Fig. 4; Supplementary Table S8). NP in Animal cup was highly abundant (96 patches ± 46.78) at all localities and sites and showed the highest abundance at the Palazzu (CorPal) site, reaching values of up to 664.33 patches ± 72.04. Animal cup exhibited the smallest MPS (values ranging from 23.10 mm^2^ ± 0.66 to 84.60 mm^2^ ± 13.83) and a high MSI (close to 0.6–0.7) (Fig. 4; Supplementary Table S8). Animal massive showed comparatively lower NP than Animal cup and Animal encrusting at all localities and sites with values ranging from 11 ± 2.94 to 153.67 ± 23.63 patches, but presented the biggest MPS with values ranging from 227.46 mm^2^ ± 144.98 to 520.40 mm^2^ ± 159.70. The MSI of Animal encrusting showed irregular shapes, with overall values below 0.5. Animal tree showed lower NP, ranging from 25.67 ± 10.69 to 180.33 ± 13.04 patches with MPS ranging from 94.97 ± 17.53 mm^2^ to 304.58 ± 51.59 mm^2^. Data showed that Animal tree was the MFG with more irregular forms, with mean values below 0.4 (Fig. 4; Supplementary Table S8).

## Discussion

Red coral populations are highly threatened by harvesting and by the dramatic effects of mortality events putatively related to climate change. However, to date the effects of these disturbances have been mainly assessed at population level^19^,^20^,^37^ and barely at community level. The present study provides the first community level, a base-line data on diversity, structure and composition patterns of coralligenous outcrops dominated by red coral.

We highlight that habitat similarity exists on a regional scale (among localities). However, strong differences in specific composition and species abundance distributions were found at site level, these differences were clearly smoothed at locality level. The most abundant species are the same among localities, evidencing the similarity of communities on a regional scale. Specifically, five out of the 15 most abundant species (most of them belonging to the Animal encrusting form) were shared in the localities studied. Interestingly, on the same regional scale, Casas-Güell *et al*.^30^ found similar consistency in composition among localities of *Paramuricea clavata* dominated assemblages. The processes behind these differential, multi-scale patterns, in structuring assemblages are difficult to infer. Species composition across localities does not appear to be determined primarily by the differences in physico-chemical conditions or differential impact of major disturbances such as mass mortality events. However, bearing in mind that sites in Catalonia and Corsica are located in marine protected areas, the shifts in structural complexity due to harvesting in Provence sites could not be discarded. Overall, however, we contend that biological factors (growth rates, recruitment, competition, successional patterns) could be the major drivers of variability found at the *Site* level (or, alternatively, should explain most of the variability found at the site level). Species structuring coralligenous outcrops generally display a limited dispersal capacity^38^,^39^,^40^ that may shape the high heterogeneity observed on a small scale. This may imply that local persistence will be enhanced once the populations are established^41^,^42^. Our results are in agreement with other studies on coralligenous assemblages, where variability observed on a small geographic scale (replicates or patches) is the highest^30^,^43^,^44^. We acknowledge that for the characterization of the assemblages expanding the number of sites per locality would be required. This would likely reduced the observed variability while would provide a broader picture of species composition and abundance in the studied assemblages. From a conservation and management perspective, it is important to adapt the monitoring schemes to encompass the variability found at small spatial scales (i.e. at site level).

This study demonstrated that morpho-functional groups tended toward common patterns when their abundances were compared on a range of regional spatial scales. The canopy was dominated by *C. rubrum* (see Supplementary Table S9) whereas basal layers were generally covered (30–50% cover) by encrusting and massive invertebrates, cup corals and a mixture-complex matrix. The Encrusting sponge was the group, with the highest number of species and was the most abundant (highest % of cover) for almost all sites and localities except Palazzu (Corsica), where the cup coral *Leptopsammia pruvoti* was the dominant group. In line with our results, high dominance and diversity of sponges have been previously reported for coralligenous outcrops^16^,^24^,^45^,^46^.

Overall, our results highlighted the high complexity and diversity of the coralligenous outcrops dominated by the red coral. Different aspects of seascape patterns (patch number, size and shape) were consistently found for all perennial species across the sites and localities studied. We showed that the assemblages were mainly characterized by moderate coverage of perennial species (e.g. sponges, anthozoans, bryozoans, and tunicates), which showed high NP, moderate MPS and complex shapes MSI. Interestingly, perennial species groups showed significant differences at site level, but usually exhibiting a seascape mainly composed of encrusting and cup forms with the highest NP. However, the largest sizes corresponded to Animal massive growth forms, followed by encrusting and tree forms. The MSI was quite irregular for all groups except for the Animal Cup, which displayed the most regular shapes. This finding may indicate that irregular forms of *encrusting*, Animal tree, and *massive species* were the most abundant in characterizing the assemblages, and they also exhibited more regular (circular) shapes when coral *cup-form* was the dominant group. Using landscape pattern indices to study spatial patterns along a depth gradient, ^8^found that coralligenous outcrops exhibited the greatest spatial pattern complexity among other benthic assemblages. The authors argued that a decrease in dynamics (% of area changed) might enhance high diversity and thus, complex spatial patterns. In light of our results, we contend that these indices may also be excellent proxies to estimate the health of coralligenous outcrops (see below). For instance, one of the most evident phenomena after disturbances in terrestrial and marine ecosystems is the significant change in number, size and shape of patches^35^,^47^,^48^. We can predict that after a large disturbance, major shifts will be observed on these parameters, from high NP, intermediated MPS and irregular forms of well-mixed groups of invertebrates, to lower values for these indices and more circular forms. However, this approach should be followed over time, and through the implementation of long-term surveys to avoid potential misunderstanding of the local variability (e.g. high natural abundance of the scleractinian cup forms with a circular shape in Palazzu, Corsica).

Overall, marine biodiversity is being eroded at unprecedented rates due to climate change and other multiple human-derived threats^49^. Coralligenous outcrops are key habitats within the Mediterranean coastal ecosystems, mainly because of their high biodiversity and structural complexity^1^. Since 2000, three different operational EU Directives: the Water Framework Directive (WFD), the Marine Strategy Framework Directive (MSFD), and the Maritime Spatial Planning Directive (MSPD) are markedly oriented to assess the environmental quality of marine ecosystems. For instance, the principal aim of the MSFD is to effectively protect the marine environment across Europe achieving Good Environmental Status (GES) of the EU’s marine ecosystems by 2020 and to protect the resource base upon which marine-related economic and social activities depend. During the recent years, different protocols and indices have been developed to gather key information for the assessment of the health status of coralligenous habitats^4^,^16^,^50^,^51^,^52^. These studies converged to assess the macrobenthic biodiversity as a key parameter to determine the ecological status of coralligenous. The fine-scale, high-resolution data presented in this study represents a step forward, as there is a need for baseline data at community level in order to plan the management of these habitats. We argue that the combinations of biotic measures used in this study are excellent metrics to measure the health of the assemblages, and to promote sound management and conservation plans for the rich coralligenous assemblages.

## Materials and Methods

### Study sites and sampling design

This study was conducted in three localities in the NW Mediterranean region (Catalonia; Provence and Corsica), covering more than 400 km^2^ (5°E-W) and more than 400 km of the coastline (Supplementary Figure S1). In the present study, sampling was assigned to a single habitat. For habitat definition, we followed Habitat Directive 92/43 EEC and considered species presence, cover, organism’s life history, water quality, and substrate among others factors^53^. We used a hierarchical, nested sampling to characterize and cope with structure and diversity variability of coralligenous outcrops dominated by the red coral *Corallium rubrum*. At each locality, three sites (separated by approximately 1 km) were sampled: in Catalonia, Cova del Dofí (CatDof), Cova de la Reina (CatRei) and Pota de Llop (CatLlo) located in the Natural Park of Montgrí, Medes Islands and BaixTer; in Provence, Ille Plane-Grotte Pérès (ProPer), Riou Sud (ProRio) and Maïre Grotte (ProMai), located in Riou Archipelago in the National Park des Calanques; and in Corsica, Palazzu (CorPal), Palazzinu (CorPlu) and Passe Palazzu (CorPas), located in the Scandola Natural Reserve (see Supplementary Table S1 for latitude/longitude coordinates).

At each site, three transects approximately 0.32 m^2^ in size (80 cm long and 40 cm wide) were sampled between depths of 15–20 m except 3 sites for which sampling was carried out between 27 and 40 m (see Supplementary Table S1). To test for the potential depth effect, we carried out an exploratory analysis testing the correlation between species distribution with depth (Relate analysis). Since no significant effect was detected (Rho = 0.088; p > 0.01), we did not consider depth as factor in the analysis (see Statistical Analysis section). No randomization of photographs was performed. Selection of the transect area was based on the results obtained from a previous study addressing the minimal sampling area (MSA) for this habitat^16^. Surveys were carried out in 2007 for most sites (6), in 2010 (1 site) and 2013 (2 sites) (see Supplementary Table S1). Previous studies on temporal changes on biodiversity assessments on coralligenous assemblages showed no significant differences over 5 years period unless strong disturbances affected the assemblages^30^,^54^. Since no major disturbances were reported in the study areas during the period 2007–2013, we did not consider time as factor in the analysis (see Statistical Analysis section). Each transect was monitored photographically using quadrats of 20 × 20 cm (400 cm^2^) to facilitate species identification. A total of 216 photographs (8 photos × 3 replicates × 9 sites) were analyzed. The photographs were taken with a Nikon D70S digital SLR camera fitted with a Nikkor 20 mm DX lens (3000 × 2000 pixel resolutions) and contained in Subal D70S housing. Lighting was achieved with two electronic strobes fitted with diffusers.

### Analysis of photographs

From each photograph, all sessile macrobenthic individuals were identified to the lowest taxonomic level (genus and species) and classified within 8 morpho-functional groups based on their taxonomy, life-span and growth form (Table 3) following^30^. Each individual patch was assigned to different species and morpho-functional groups.

For each MFG we calculated the diversity (number of perennial species) and the abundance (measured as % cover). For the Animal tree morphofunctional group (e.g. *Corallium rubrum*), cover was measured as the surface attached to the substrate as our main objective was to estimate the cover of each species in the whole assemblage.to characterize the structural complexity. Finally, for the overall set of perennial species, which are characterized by high longevity and slow population dynamics (see Appendix II), we also quantified the spatial configuration using the number of patches (NP), the mean patch size (MPS: mm^2^) and the mean shape index (MSI) using the Seascape software^55^. MSI was calculated applying the following formula^56^:


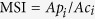


where Ap is the patch area of the patch *i* and Ac_i_ is the area of a perfect circle with perimeter equivalent to the patch *i*. This indices measures patch circularity. A value of 1 represents a perfect circle, while 0 is approached when the outline of the patch becomes irregular. The relevance of these three indices (NP, MPS and MSI) to detect spatial seascape patterns in Mediterranean rocky communities was evaluated previously^33^,^57^.

Spatial patterns of diversity metrics for perennial species were assessed by quantifying the number of species, which is the average number of species per sampling unit; and beta diversity, which corresponds to the multivariate distance between group-centroids determined using the PERMDISP procedure. PERMDISP is employed to compare the degree of multivariate dispersion of different groups of samples based on a distance matrix. When PERMDISP is used on a Jaccard distance presence/absence matrix, it is directly interpretable as a test for similarity in beta diversity among groups^58^. Additionally, the pool of species at locality spatial level or gamma diversity (i.e. the number of species observed within the sites at each locality studied) was calculated.

### Statistical analyses

The variability of structural complexity of assemblages, both in terms of morpho-functional groups and specific composition of perennial species assemblages, was tested based on the hierarchical sampling design. It included two spatial factors: *Locality* (random factor, three levels) and *Site* (random factor, three levels, nested in *Locality*). A non-parametric analysis of variance, PERMANOVA^59^, was applied using Bray-Curtis and Euclidean distances for multivariate and univariate analyses, respectively.

To visualize similarity patterns at different spatial scales (e.g. *Site* and *Locality*), a non-metric multi-dimensional scaling (nMDS) ordination analysis was performed based on the Bray–Curtis similarity measure for presence/absence and abundance (cover %) data. Furthermore, a similarity percentage analysis (SIMPER^60^) was performed to identify the relative contribution of each taxa and each MFG to the significant dissimilarities among sites and localities.

Statistical analyses were computed using the program Primer v6 with the PERMANOVA + add-on package.

## Additional Information

**How to cite this article**: Casas-Güell, E. *et al*. Structure and biodiversity of coralligenous assemblages dominated by the precious red coral *Corallium rubrum* over broad spatial scales. *Sci. Rep.* **6**, 36535; doi: 10.1038/srep36535 (2016).

**Publisher’s note**: Springer Nature remains neutral with regard to jurisdictional claims in published maps and institutional affiliations.

## Supplementary Material

Supplementary Information

## Figures and Tables

**Table 1:** Species by morpho-functional groups contributing more than 50% to the similarity of studied regions.

	Similarity		
	Provence (61.51%)	Corsica (45.51%)	Catalonia (38.78%
Animal encrusting	*Pleraplysilla spinifera* (35.35%)*Crella (Grayella) pulvinar* (8.65%)Encrusting sponge n.idd. (4.57%)*Scalarispongia scalaris* (4.36%)Serpulidae (1.69%)	*Pleraplysilla spinifera* (9.36%)Encrusting bryozoans n.idd. (15.35%)Encrusting sponge n.idd. (7.01%)*Gregarinidra gregaria* (6.17%)	*Pleraplysilla spinifera* (9.97%)Encrusting sponge n.idd. (13.50%)Encrusting bryozoans (6.28%)
Animal massive	*Oscarella* sp.(5.88%)*Petrosia ficiformis* (2.71%)*Ircinia variabilis* (2.40%)*Aplysina cavernicola* (2.27%)	*Haliclona mucosa* (4.78%)	*Petrosia ficiformis* (5.27%)
Animal tree	*Corallium rubrum* (17.71%)	*Corallium rubrum* (3.91%)*Reteporella grimaldii* (3.75%)	*Corallium rubrum* (11.89%)
Animal cup	*Caryophyllia inornata* (2.89%)*Hoplangia durotrix* (2.45%)	*Leptopsammia pruvoti* (30.78%)	*Leptopsammia pruvoti* (8.87%)

The underlined species are those contributing to the similarity of most of the sites. Similarity analysis based on % cover dataset. The average similarity for the NW Mediterranean was 35.45%.

**Table 2:** Species by morphofunctional groups contributing more than 50% to the dissimilarity of studied regions.

Dissimilarity			
	Provence VS Corsica (76.78%)	Provence VS Catalonia (66.01%)	Catalonia VS Corsica (68.38%)
Animal encrusting	*Pleraplysilla spinifera* (12.57%)*Gregarinidra gregaria* (9.04%)Encrusting bryozoan n.idd. (4.61%)*Crella (Grayella) pulvinar* (4.55%)	*Pleraplysilla spinifera* (14.60%)*Scalarispongia scalaris* (4.31%)*Crella (Grayella) pulvinar* (3.89%)*Dendroxea lenis* (3.84%)*Parazoanthus axinellidae* (3.35%)	*Gregarinidra gregaria* (11.32%)*Pleraplysilla spinifera* (6.19%)Encrusting bryozoans (4.81%)*Haliclona (Sostella) mucosa* (3.62%)Sponge n.idd. (3.87%)*Parazoanthus axinellae* (3.56%)*Crambe crambe* (3.36%)*Dendroxea lenis* (3.33%)
Animal massive	*Oscarella sp.* (4.59%)	*Petrosia ficiformis* (5.24%)*Oscarella spp.* (4.54%)	*Petrosia ficiformis* (5.18%)
Animal cup	*Leptosammia pruvoti* (10.02%)	—	*Leptosammia pruvoti* (9.42%)

The underlined species are those contributing to the similarity of most of the sites. Analysis based on cover (%) dataset.

**Table 3:** Morphofunctional groups.

Biological categories	Description
Seasonal algal turf	Annual erect or semi-erect fleshy algal species, with one or multiple zones of attachment to the substratum; generally constitutes algal cushions or thin sheets with mixtures of algal species.
Seasonal animal turf	Small seasonal animal species, mainly bryozoans and hydrozoans; usually is forming animal cushions or thin sheets with mixtures species.
Seasonal mixture complex turf	Small seasonal algae and animal species (mainly bryozoans and hydrozoans), sediment, detritus and fragments; normally forming cushions or thin sheets with mixtures of species.
Perennial algal encrusting	Species growing mainly as two dimensional sheets; more or less completely attached to the substratum.
Perennial algal erect	Species attached to the substratum usually with a unique zone (visible even in winter) of basal attachment to the substratum.
Perennial algal turf – invasive	Perennial dense thick filamentous turf algae with the ability to maintain permanent carpets (e.g. the invasive species *Womersleyella setacea*).
Perennial animal encrusting	Species of sponges, cnidarians, bryozoans and tunicates growing as two dimensional sheets; more or less completely attached to the substratum.
Perennial animal massive	Mound species of sponges and cnidarians with vertical and lateral growth; normally attached to the substratum all along their basal area.
Perennial animal tree	Erect species of cnidarians and bryozoans, more or less branched; usually with a single point of attachment to the substratum.
Perennial animal cup	Solitary corals attached to the substratum all along their basal area.
Perennial animal boring	Excavating organisms living into the rock (e.g. *Cliona viridis*).
Perennial animal epibiont	Species growing over other invertebrates or calcareous algae (mainly polychaetes e.g. *Salmacina dysteri* or *Filograna implexa* and bryozoans e.g. *Chartella tenella*).

Biological categories used in this study combining taxonomy, life span and morphological growth forms (adapted from Garrabou *et al*. 2002 and Teixidó *et al*. 2011).
