# Supplementary material for: Structure and biodiversity of coralligenous assemblages dominated by the precious red coral Corallium rubrum over broad spatial scales
Source: Sci Rep. 2016 Nov 18;6:36535. doi: 10.1038/srep36535 (PMC5114658; doi:10.1038/srep36535)
Supplement: Supplementary Information [file srep36535-s1.pdf]

Supplementary information

Structure and biodiversity of coralligenous assemblages dominated by the precious red coral *Corallium rubrum* over broad spatial scales

Edgar Casas-Güell*[1], Emma Cebrian[2,3], Joaquim Garrabou[1,4], Jean-Baptiste Ledoux[5,1], Cristina Linares[6], Núria Teixidó[7]

[1]Institut de Ciències del Mar (ICM-CSIC), Passeig Marítim de la Barceloneta 37-49, 08003 Barcelona, Spain

[2]Centre d'Estudis Avançats de Blanes (CEAB-CSIC), Accés Cala Sant Francesc 14, 17300 Blanes, Girona, Spain

[3]Departament de Ciències Ambientals, Facultat de Ciències, Universitat de Girona, Girona, Spain

[4]UM110, CNRS/INSU, IRD, Aix-Marseille Université, Université du Sud Toulon Var, Mediterranean Institute of Oceanography (MIO), Marseille, France

[5]CIMAR/CIIMAR, Centro Interdisciplinar de Investigaçao Marinha e Ambiental, Universidade do Porto, Rua dos Bragas 177, 4050-123 Porto, Portugal

[6]Departament d´Ecologia, Facultat de Biologia, Universitat de Barcelona, Avinguda Diagonal 643, 08028, Barcelona, Spain

[7]Stazione Zoologica Anton Dohrn, Villa Dohrn-Benthic Ecology Center, Punta San Pietro, Ischia, Naples 80077, Italy

Supplementary Figure S1. **Geographic area**. Map showing the Mediterranean geographic area where the photographic surveys were conducted. Map created with R program “maps” package (R Development Core Team (2008). R: A language and environment for statistical computing. R Foundation for Statistical Computing, Vienna, Austria. ISBN 3-900051-07-0, URL http://www.R-project.org.)

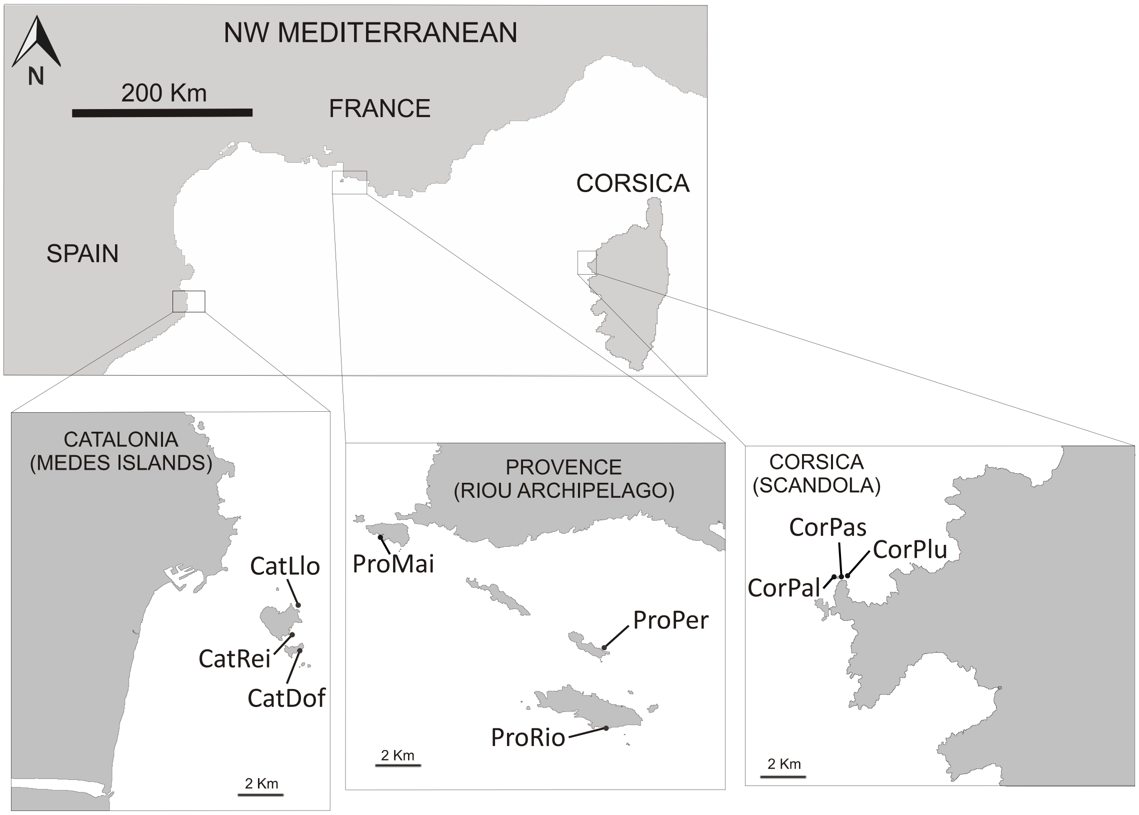

Supplementary Figure S2. Cover % values (mean ± SD) for benthic categories for each site studied of the NW Mediterranean region.

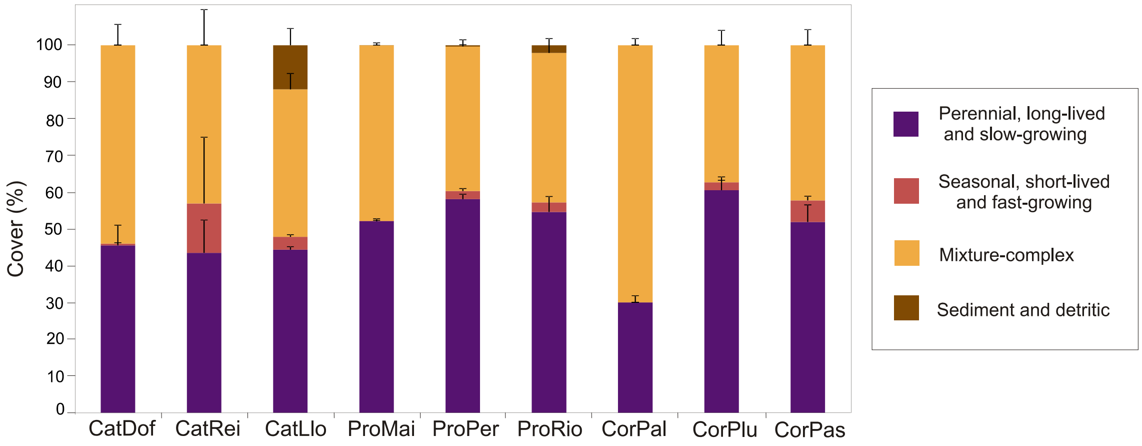

Supplementary Figure S3. Landscape pattern indices. Number of patches (NP± SD), Mean Patch Size (MPS± SD) and Mean Shape Index (MSI± SD) for the overall set of perennial species.

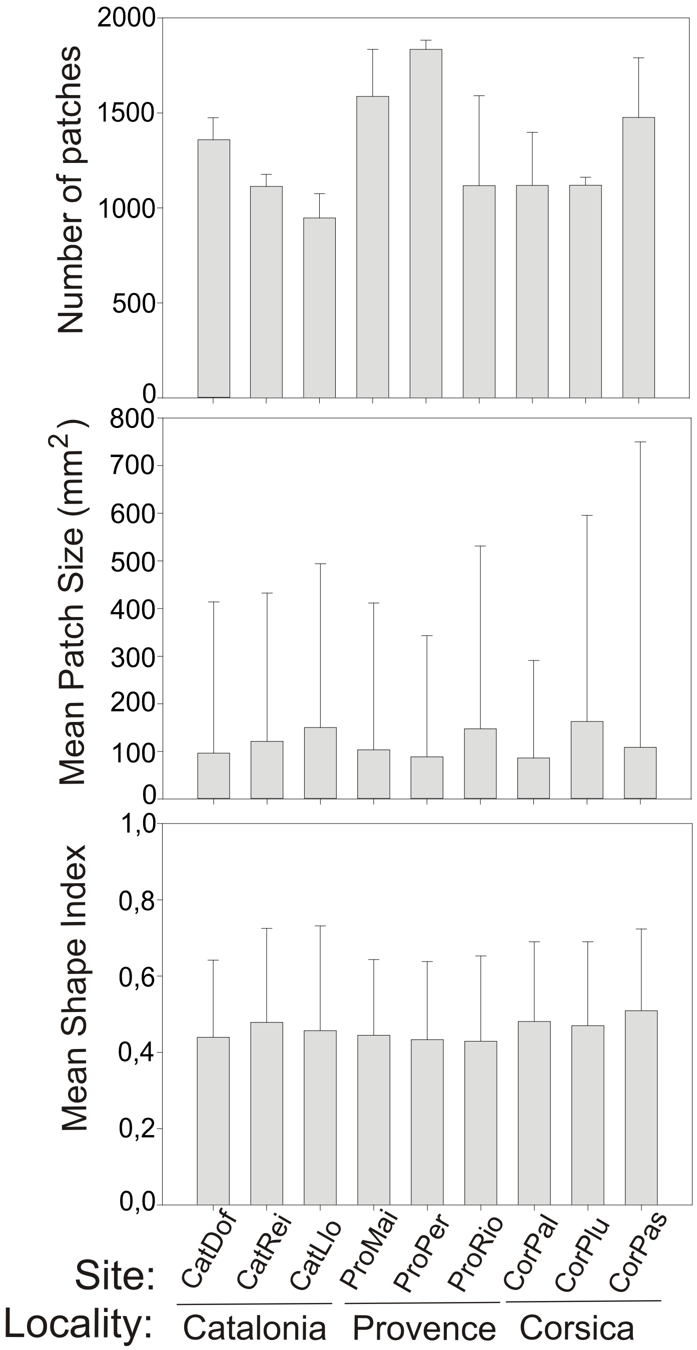

Supplementary Table S1. Latitude/longitude for the sites studied.

Site	Locality	Codes	Latitude N	Longitude E	Year	Depth
Cova del Dofí	Catalonia	CatDof	42° 2’ 51.07”	3° 13’ 31.73”	2007	15
Cova de la Reina	Catalonia	CatRei	42° 2’ 46.14”	3° 13’ 29.03”	2007	20
Pota del Llop	Catalonia	CatLlo	42° 2’ 58.20”	3° 13’ 31.94”	2013	30
Maïre Grotte	Provence	ProMai	43°12’ 36.72”	5° 19’ 57.83”	2007	18
Plane Grotte Pérès	Provence	ProPer	43°11’ 12.48”	5° 23’ 25.04”	2007	15
Riou Grotte Sud	Provence	ProRio	43°10’ 22.44”	5° 23’ 21.88”	2007	20
Palazzu	Corsica	CorPal	42° 22’ 48.72”	8° 32’ 44.70”	2007	20
Palazzinu	Corsica	CorPlu	42° 22’ 47.71”	8° 33’ 0.90”	2013	40
Passe Palazzu	Corsica	CorPas	42° 22’ 47.64”	8° 32’ 51.29”	2010	27

Supplementary Table S2. List of the taxa surveyed in the three studied localities of the NW Mediterranean.*Morphological growth form*: encrusting (Enc), tree (Tree), cup (Cup), massive (Mas), turf (Turf), epibiont (Epi). *Life span*: seasonal (Sea), perennial (Per). SIMPER perennial species dataset: species that belong to similarity group of 50% of cumulative contribution in similarity percentage analysis (denoted by *) and presence by +; CatDof = 68.28%; CatRei = 63.02%; CatLlo = 62.43%; ProPer = 77.22%; ProRio = 66.13%; ProMai = 70.57%; CorPal = 68.23%; CorPas = 65.96%; CorPlu = 70.14%.

Taxa	Growth form	Lifes pan	Catalonia			Provence			Corsica		
			**CatDof**	**CatRei**	**CatLlo**	**ProRio**	**ProMai**	**ProPer**	**CorPas**	**CorPlu**	**CorPal**
**Clorophyta**											
Palmophyllumcra ssum	Enc	Per		+						+	
Flabellia petiolata	Ere	Per	+								
**Rhodophyta**											
Lithophyllum cabioache/stictaeforme	Enc	Per		+							
Mesophyllum alternans	Enc	Per		*	+	*				*	
Peyssonnelia sp.	Enc	Per		*	+	+				*	
Red encrusting	Enc	Per		+		+		+			
**Mixture complex**	Turf	Sea/Per	+	+	+	+	+	+	+	+	+
**Protozoa**											
Miniacina miniacea	Enc	Per		+		+	+	+	+	+	+
**Porifera**											
Acanthella acuta	Mas	Per		+	+			+			
Agelas oroïdes	Mas	Per	+	*	+			+	+	+	
Aplysina cavernicola	Mas	Per					*	*		+	
Aplysilla sulfurea	Enc	Per		+	+	+	+	+	+		
Axinella damicornis	Mas	Per	+	+	*	+	+	+	+	+	+
Chondrosia reniformis	Mas	Per			+					+	
Clathrina sp.	Mas	Per	+	*	+	+			+	+	
Cliona sp.	Bor	Per			+			+			
Corticium candelabrum	Mas	Per	+	+	+						
Crambe crambe	Enc	Per		+	*	+	+	+	+	+	
Crella pulvinar	Enc	Per	+	*	*	*	*	*	+	+	
Dendroxea lenis	Enc	Per	*	+	+	+	+	*	+	*	*
Diplastrella bistellata	Enc	Per	*	+							
Fasciospongia cavernosa	Enc	Per		+	+	+	+	+		+	
Haliclona fulva	Enc	Per	+	*	+	+	+	+		*	*
Haliclona mediterranea	Enc	Per				+				+	+
Haliclona mucosa	Enc	Per	*	+	+		+	*	*	*	+
*Hymedesmia*_type	Enc	Per								+	
Hemimycale columella	Enc	Per			+	+		+			
Hexadella pruvoti	Enc	Per	+	+					+		
Hexadella racovitzai	Enc	Per	+		+	+			+		
Ircinia fasciculata	Mas	Per	+								
Ircinia variabilis	Mas	Per	+	*	+	*	*	+	+	*	+
Ircinia oros	Mas	Per	+	*	+	+	+	+			+
Oscarella sp.	Mas	Per	+	*	+	*	*	+			+
Petrosia fisciformis	Mas	Per	*	+	+	+	*	*	+	+	*
Phorbas tenacior	Enc	Per	+	*	+		+	+		+	
Phorbas topseti/fictitius	Enc	Per				+					
Plakina sp.	Enc	Per				+		+	+	+	
Plakortis sp.	Enc	Per					+				+
Pleraplysilla spinifera	Enc	Per	+	*	*	*	*	*	*	*	*
Prosuberites longispinus	Enc	Per		*		+	+	*	+	*	+
Raspaciona aculeata	Enc	Per		+	+			+		+	
Sarcotragus muscarum	Mas	Per	+							+	
Sarcotragus foetidus	Mas	Per			+	+	+				
Scalarispongia scalaris	Enc	Per	+	+	*	*	+	*	+	+	
Spongia officinalis	Mas	Per						+			
Spongia virgultosa	Mas	Per					+	+			
Spirastrella cunctatrix	Enc	Per		+	+		+			+	
Terpios granulosa	Enc	Per		+		+	+	+	+	+	
Other sponge	Enc/ Mas	Per	*	*	*	*	*	*	+	*	*

**Anthozoa**														
Alcyonium acaule	Mas	Per			+	+								
Alcyonium coralloides	Enc	Per	+		+									
Caryophyllia inornata	Cup	Per	+	+	+	+		*	*		+	+		+
Corallium rubrum	Tree	Per	*	*	*	*		*	*		*	+		*
Corynactis viridis	Mas	Per				+								
Eunicella cavolinii	Tree	Per						+						
Hoplangia durotrix	Cup	Per	*	+	+	+		*		+	+	+		*
Leptopsammia pruvoti	Cup	Per	*	*	*	+				+	*	*		*
Paramuricea clavata	Tree	Per			*	+						+		
Parazoanthus axinellae	Enc	Per		+	*									
Parerythropodium coralloïdes	Enc	Per				+								
**Polychaeta**														
Filograna implexa/*Salmacina dysteri*	Epi	Per	+		+	+					+	+	+	
Protula sp./*Serpula vermicularis*	Enc	Per	+	+	+	+	+			*	+	+	+	
Serpulidae	Enc	Per	*	+	+	+	+			*	*	+		
**Mollusca**														
Bivalvia	Enc	Per				+				+				
**Bryozoa**														
Adeonella calveti/Smittina	Tree	Per			*									
cervicornis										+	+	+		
Beania magellanica	Enc	Per										+		
Celleporina caminata	Epi	Per				+	+			*				
Chartella sp.	Epi	Per			*	+				+	+			+
Dentiporella sardonica	Enc	Per						+						
Disporella hispida	Turf	Sea			+	+		+		+				
Encrusting Bryozoans	Enc	Per	*	+	*	+		+		*	*	*		*
Gregarinidra gregaria	Enc	Per									*	+		
Myriapora truncata	Tree	Per		+	+							+		
Reteporella grimaldii	Tree	Per			+	+				+	*	*		+
Rhynchozoon sp.	Enc	Per			+	+		+		+				
Schizomavella linearis	Enc	Per			+			+		+				
Turbicellepora sp	Epi	Per										+		
**Tunicata**														
Aplidium sp.	Enc	Per			+			*		+		+		+
Cystodites dellechiajei	Mas	Per	*		*									
Didemnidae	Enc	Per			+			+		+	+	+	+	
Didemnum coriaceum	Enc	Per									+			
Halocynthia papillosa	Mas	Per	+		+									

Supplementary Table S3. Diversity measures at different hierarchical spatial scales: alpha diversity (α-diversity ± SD) % of unshared species (β-diversity) and local diversity (γ–diversity).

Site and locality	α	β (Unshared species %)	Gamma diversity
**Catalonia**		34.45± 1.78	68
Dofí	24 ± 2	17.81 ± 3.66	
Reina	33 ± 0.58	20.03 ± 0.68	
PotaLlop	41 ± 5.29	20.61 ± 1.65	
**Provence**		31.10± 1.61	72
Maïre	30 ± 5.78	21.60 ± 0.77	
Planes Pérès	39 ± 3.2	19.43 ± 1.34	
Riou sud	34 ± 3.2	24.00 ± 1.33	
**Corsica**		32.21± 1.12	57
Palazzinu	40 ± 3.1	14.93 ± 1.48	
Palazzu	21 ± 4.2	17.40 ± 1.39	
PassePalazzu	26 ± 1.53	17.69 ± 1.19	

Supplementary Table S4. PERMANOVA analysis on the number and % of cover of perennial species of the morpho-functional groups on the basis of Bray-Curtis dissimilarity measure.

	Source of variation	df	SS	MS	Pseudo-F	P(perm)
Number of species	Locality	2	236.07	118.04	1.6344	0.2492
	Site (Locality)	6	433.33	72.222	8.945	0.0001
	Residual	18	145.33	8.0741		
	Total	26	814.74			
Cover %	Locality	2	3884.4	1942.2	1.4787	0.2275
	Site (Locality)	6	7880.7	1313.4	12.538	0.0001
	Residual	18	1885.6	104.76		
	Total	26	13651			

Supplementary Table S5. PERMANOVA analyses of community structure and specific composition on the basis of Bray-Curtis dissimilarity measure.

Abundance	Source of variation	df	SS	MS	Pseudo-F	P(perm)	ECV (Sq. root)
Cover (%)	Locality	2	22015	11007	2.5998	0.0116	27.434
	Site (Locality)	6	25403	4233.9	7.7889	0.0001	35.073
	Residual	18	9784.5	543.58			23.315
	Total	26	57202				
Presence and absence	Locality	2	7645.5	3822.7	2.4464	0.0105	15.847
	Site (Locality)	6	9375.6	1562.6	7.0219	0.0001	21.135
	Residual	18	4005.6	222.53			14.917
	Total	26	21027				

Supplementary Table S6. Univariate analysis of variance for alpha diversity with permutation tests (PERMANOVA) based on Euclidian distances. Beta diversity (% unshared species) tested with PERMDISP routine.

Diversity metrics	Source	df	SS	MS	Pseudo-F	P-value
Species number	Locality	2	156.22	78.11	0.39591	0.6709
	Site (Locality)	6	1183.8	197.3	18.626	0.0001
	Residual	18	190.67	10.60		
	Total	26	1530.7			
Beta diversity (% unshared species)	Source	df1	df2	F	P-value	
	Locality	2	24	1.2974	0.3316	
	Site	8	18	2.7976	0.2285	

Supplementary Table S7. Univariate PERMANOVA analyses of Number of patches (NP), Mean patch size (MPS) and Mean shape index (MSI) for overall set of perennial species on the basis of Euclidian distances.

Seascapepatternindices	Source of variation	df	SS	MS	Pseudo-F	P(perm)	ECV (Sq. root)
Number of patches	Locality	2	2.27e5	1.14e5	1.79260	0.2930	38.612
	Site (Locality)	7	4.40e5	62805	0.63582	0.7130	-58.421
	Residual	18	9.68e6	98778			314.29
	Total	98	1.02e7				
Mean patch size	Locality	2	1.6588	0.82938	1.1936	0.343	6.3167e-2
	Site (Locality)	6	4.6673	0.66668	0.2837	0.952	-0.39972
	Residual	98	230.37	2.3507			1.5332
	Total	107	237.19				
Mean shape index	Locality	2	1.28e-2	6.38e-3	0.6365	0.557	-1.0401e-2
	Site (Locality)	6	7e-2	1e-2	0.8455	0.556	-1.3169e-2
	Residual	98	1.1591	1.18e-2			0.10876
	Total	107	1.2507				

Supplementary Table S8. Univariate PERMANOVA analyses of Number of patches (NP), Mean patch size (MPS) and Mean shape index (MSI) for each morphofunctional group at Site and Locality spatial levels.

Seascape Pattern indices	Morfofunctional group	Source of variation	*df*	SS	MS	*Pseudo-F*	*p-value*	ECV (Sq.root)
NP	Encrusting	Locality	2	9.82e5	4.91e5	2.972	0.1215	196.61
		Site	7	1.17e6	1.67e5	3.821	0.0102	216.47
		(Locality)	17	7.44e5	4377			209.21
		Residual	26	2.64e6				
		Total						31.201
	Massive		2	22783	1139	3.574	0.0782	31.785
		Locality	7	22624	3232	5.671	0.0011	23.872
		Site	17	9688	569.9			
		(Locality)	26	56275				37.691
		Residual						45.884
	Tree	Total	2	37520	18760	2.764	0.1355	36.513
			7	48164	880.6	5.161	0.004	
		Locality	17	22665	333.2			113.79
		Site	26					147.48
		(Locality)						93.13
	Cup	Residual	2	3.48e5	1.74e5	2.678	0.1442	
		Total	7	4.61e5	65986	7.608	0.0004	
			17	1.47e5	8673.1			
		Locality	26					
		Site						
		(Locality)						
		Residual						
		Total						
MPS	Encrusting	Locality	2	0.986	0.493	0.558	0.5823	-0.2154
		Site (Locality)	7	6.276	0.897	6.063	0.0014	0.5331
		Residual	17	2.514	0.148			0.3846
		Total	26	9.563				
	Massive	Locality	2	1.5170.758		0.191	0.8099	-0.617
		Site(Locality)	7	28.124.018		2.634	0.0474	0.9726
		Residual	17	25.931.523				1.2351
		Total	26	55.80				
						1.868		0.3228
	Tree	Locality	2	3.7811.891		3.375	0.221	0.5231
		Site(Locality)	7	7.1711.024			0.018	0.5509
		Residual	17	5.1600.304				
		Total	26	17.12				
						1.1048		0.0025
	Cup	Locality	2	1.1070.0053		8.5358	0.4038	0.1287
		Site(Locality)	7	0.3460.0049			0.0002	0.0076
		Residual	17	0.0100.0006				
		Total	26	0.552				
MSI	Encrusting	Locality	2	3.3e-4	1.6e-4	6.3e-2	0.9315	-1.70e-2

	Site (Locality)	7	1.8e-2	2.6e-3	2.79	0.0394	2.528e-2
	Residual	17	1.6e-2	9.4e-4			3.071e-2
	Total	26	3.5e-2				
					1.57		
Massive	Locality	2	8.1e-3	4.07e-3	2.71	0.302	1.3e-2
	Site(Locality)	6	1.8e-2	2.63e-3		0.045	2.5e-2
	Residual	18	1.6e-2	9.69e-4	0.15		3.1e-2
	Total	26	4.9e-2		6.63		
						0.8763	
Tree	Locality	2	1.38e-3	6.9e-4	1.27	0.0006	-2.13e-2
	Site(Locality)	6	3.20e-2	4.6e-3	11.49		3.84e-2
	Residual	18	1.17e-2	6.9e-4		0.3518	2.62e-2
	Total	26	4.50e-2			0.0001	
			1.78e-2	8.9e-3			1.50e-2
Cup	Locality	2	4.96e-2	7.1e-3			4.96e-2
	Site(Locality)	6	1.05e-2	6.2e-4			2.48e-2
	Residual	18	8.21e-2				
	Total	26					

Supplementary Table S9. Abundance of *Corallium rubrum* colonies expressed as density (number of colonies / m^2).

Site	Mean ± SE	Minimum	Maximum	Density
Pota Llop	30.67 ± 3.39	19	42	95.83
Cova Dofi	77.33 ± 3.05	67	85	241.67
Cova Reina	83.67 ± 5.65	60	120	261.46
Riou sud	77.67 ± 5.42	45	102	242.71
Plane Grotte Peres	177 ± 4.48	158	198	553.13
Maïre Grotte	134 ± 6.18	104	177	418.75
Passe Palazzu	38 ± 3.57	27	52	118.75
Palazzinu	13 ± 3.23	6	25	40.63
Palazzu	19.67 ± 1.07	19	21	61.46
